# Emergency anticancer therapy in intensive care medicine: A mainland China survey

**DOI:** 10.62838/jccm-2026-0019

**Published:** 2026-07-27

**Authors:** Hao Zhang, DongHao Wang, ChangSong Wang, Xue-Zhong Xing

**Affiliations:** National Cancer Center/National Clinical Research Center for Cancer, Cancer Hospital, Chinese Academy of Medical Sciences and Peking Union Medical College, Beijing, China; Tianjin Medical University Cancer Institute & Hospital, Tianjin, China; First Affiliated Hospital of Harbin Medical University, Harbin, China

**Keywords:** emergency anticancer therapy, intensive care unit, survey

## Abstract

**Objective:**

To identify intensivists‘ attitudes toward emergency anticancer therapy (EAT) in patients with cancer in intensive care units (ICUs).

**Material and methods:**

A 11-question survey was performed among intensivists in the Cancer Critical Care Medicine Committee of the Chinese Anti-Cancer Association and Critical Care Medicine Committee of Beijing Association of Oncology between November 11 and December 11, 2024. Response were compared between mixed and oncologic ICU physicians.

**Results:**

In total, 120 intensivists completed the survey. For 9 of 11 questions, the agreement rate exceeded 85%. There were 61.5% mixed ICU physicican and 35.5% oncologic ICU physicians in favor of compostion of the composition of a multidisciplinary team (MDT) composing of an intensivist, an oncologis and a pharmacist, respectively. However this difference was not significant (P=0.281).

**Conclusions:**

Intensivists in China generally hold positive attitudes toward emergency anticancer therapy in ICUs for critically ill patients with cancer-related organ dysfunction. However, opinions differ regarding the MDT composition between mixed and oncologic ICU physicicans.

## Introduction

Data from cohort studies suggest that administering cancer chemotherapy alongside life-sustaining therapies in critically ill patients with cancer-related organ dysfunctions, such as organ or vessel compression, tissue infiltration, tumor lysis syndrome, is feasible and associated with meaningful survival benefits in selected patients [1]. These benefits are mostly seen in patients with hematological malignancies, as reflected in consensus and review [2–3]. Moreover, patients with solid tumors who received chemotherapy have also demonstrated improved long-term survival [4]. Notably, cancer treatment could be resumed in 58% of ICU survivors with aggressive hematological malignancies [5], indicating that emergency anticancer therapy (EAT) in intensive care medicine improves short term outcomes and potentially prolongs the overall survival of patients who had the opportunity to receive further cancer treatment. Therefore, we surveyed to identify intensivists’ attitude towards EAT for patients with cancer in intensive care units (ICUs).

## Material and methods

We surveyed ntensivists at the Cancer Critical Care Medicine Committee of the Chinese Anti-Cancer Association and Critical Care Medicine Committee of Beijing Association of Oncology between November 11 and December 11, 2024. The participants completed the survery through an online survey software (Survery Star). The survey included 11 questions (Table.1). All questions were designed based on recent reviews and consensus, demonstrating survival benefit of EAT in selected hematological and solid tumor patients [2–3]. The responders were instructed to instructed to share their perspective about EAT.

**Table 1.** Eleven questions about emergency anticancer therapy in oncologic patients

**No.**	**Question**
1	Is EAT in intensive care medicine feasible
2	Is EAT ever performed in your department
3	Is EAT for lymphoma under organ supporting therapy feasible
4	Is EAT for small cell lung cancer under organ supporting therapy feasible
5	Is EAT for non-small cell lung cancer with positive driver gene under organ supporting therapy feasible
6	Is Emergency radiotherapy for lung cancer under organ supporting therapy feasible
7	Is EAT for lymphoma with tumor lysis syndrome feasible
8	Is EAT for lymphoma with tumor-associated hemophagocytic lymphohistiocytosis or disseminated intravascular coagulation feasible
9	Is EAT for lymphoma with hyperleukocytosis feasible
10	Is EAT for lymphoma with plasma hyperviscosity syndrome feasible
11	Organization of multidisciplinary team of EAT
	Intensivist and oncologist
	Intensivist, oncologis and pharmacist
	Intensivist, oncologist, pharmacist and member of the Ethics Committee
	Intensivist, oncologist, pharmacist, member of the Ethics Committee, and relatives

EAT, emergency anticancer therapy

### Statistical analysis

Answers to the survery questions were selected from among one of three answers (ie. Yes, No, and Not sure). For the last questions (Organization of multidisciplinary team of EAT), the answers were selected from opinions. All variables were categorical, and the adjusted χ^2^ test was used to determine whether difference existed between the mixed ICU and oncologic ICU. p < 0.05 was considered indicate significance.

## Results

**Fig. 1.**
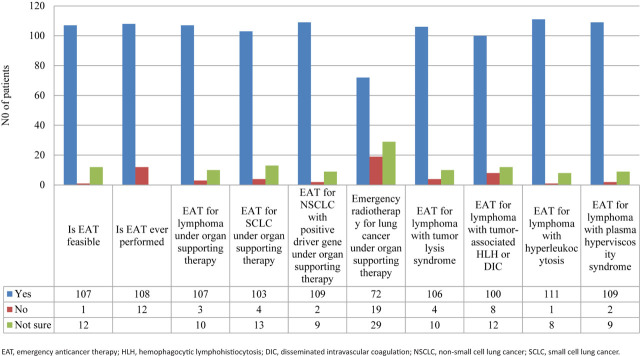
Main findings of 10 questions about emergency anticancer therapy.

In total, 120 intensivists completed the 11-question survey. The main findings are listed in Figure 1.

Notably, 89.2% (107/120) believe that EAT is feasible and 90% (108/120) reported implementing EAT in the wards. The majority (107/120,89.2%) reported that they would perform EAT in patients with lymphoma who require organ suppor, such as ventilation support for acute respiratory insufficiency, or blood purification for acute kidney injury. Similarly, 85.8% (103/120) stated that they would perform EAT in patients with small cell lung cancer and acute respiratory insufficiency under ventilation support. Furthermore, (90.8% intensivists (109/120) reported that they would provide target therapy to patients with lung cancer harboring driver gene mutations and acute respiratory insufficiency requiring ventilation support. In contrast, only 60% (108/120) considered emergency radiotherapy appropriate in patients with lung cancer and acute respiratory insufficiency under ventilation support. Most intensivists agreed with a recent review [2], reporting willingness to administer EAT in cases of tumor lysis syndrome (106/120,88.3%), disseminated intravascular coagulation or malignancy-associated hemophagocytic lymphohistiocytosis (100/120,83.3%), hyperleukocytosis (111/120,92.5%), and plasma hyperviscosity syndrome (109/120,90.8%) in patients with hematologic malignancies. However, opinions differed regarding the organization of the multidisciplinary team (MDT). Of 120 intensivists, 26(21.6%) believed that decision-making could be handled through consultation between an intensivist and oncologist, 46 (38.3%) favored including a pharmacist as well, and 46 (38.3%) advocated for an MDT comprising an intensivist, an oncologist, a pharmacist, and a member of the Ethics Committee. Finally, when attitudes woward EAT were compared between mixed ICU and oncologic ICU physicians, no significant differences were observed (Table 2).

**Table 2.** Attitudes towards emergency anticancer therapy between mixed and oncologic ICU physicians

**Question**	**Mixed ICU physicians (n =13)**	**Oncologic ICU (n =107)**	**χ^2^**	**P value**
Is EAT feasible			0.578	0.749
Yes	11 (84.6)	96 (89.7)		
No	0 (0.0)	1 (0.9)		
Not sure	2 (15.4)	10 (9.3)		

Is EAT ever performed			0.038	0.493
Yes	11 (84.6)	97 (90.7)		
No	2 (15.4)	10 (9.3)		

EAT for lymphoma under organ supporting therapy			0.387	0.824
Yes	12 (92.3)	95 (88.8)		
No	0 (0.0)	3 (2.8)		
Not sure	1 (7.7)	9 (8.4)		

EAT for small cell lung cancer under organ supporting therapy			0.689	0.708
Yes	12 (92.3)	91 (85.0)		
No	0 (0.0)	4 (3.7)		
Not sure	1 (7.7)	12 (11.2)		

EAT for non-small cell lung cancer with positive driver gene under organ supporting therapy			1.471	0.479
Yes	13 (100.0)	96 (89.7)		
No	0 (0.0)	2 (1.9)		
Not sure	0 (0.0)	9 (8.4)		

Emergency radiotherapy for lung cancer under organ supporting therapy			1.771	0.412
Yes	10 (76.9)	62 (57.9)		
No	1 (7.7)	18 (16.8)		
Not sure	2 (15.4)	27 (25.2)		

EAT for lymphoma with tumor lysis syndrome			1.925	0.382
Yes	13 (100.0)	93 (86.9)		
No	0 (0.0)	4 (3.7)		
Not sure	0 (0.0)	10 (9.3)		

EAT for lymphoma with tumor-associated hemophagocytic lymphohistiocytosis or disseminated intravascular coagulation			3.122	0.210
Yes	11 (84.6)	89 (83.2)		
No	2 (15.4)	6 (5.6)		
Not sure	0 (0.0)	12 (11.2)		

EAT for lymphoma with hyperleukocytosis			0.144	0.930
Yes	12 (92.3)	99 (92.5)		
No	0 (0.0)	1 (0.9)		
Not sure	1 (7.7)	7 (6.5)		

EAT for lymphoma with plasma hyperviscosity syndrome			3.238	0.198
Yes	11 (84.6)	98 (91.6)		
No	1 (7.7)	1 (0.9)		
Not sure	1 (7.7)	8 (7.5)		

Organization of multidisciplinary team			3.822	0.281
Intensivist and oncologist	1 (7.7)	25 (23.4)		
Intensivist, oncologis and pharmacist	8 (61.5)	38 (35.5)		
Intensivist, oncologist, pharmacist and member of the Ethics Committee	4 (30.8)	42 (39.3)		
Intensivist, oncologist, pharmacist, member of the Ethics Committee, and relatives	0 (0.0)	2 (1.9)		

EAT, emergency anticancer therapy; ICU, intensive Care Unit.

## Discussion

In this study, we reported two main findings. First, there is a general positive attitude toward EAT in intensive care medicine for critically ill patients with cancer-related organ dysfunctions. Second, the attitudes regarding EAT did not differ between mixed and oncologic ICU physicians.

Darmon et al. first reported a cohort of 100 patients with new diagnosed malignancies who received immediate chemotherapy in the ICU. Among them, 80% had hematological malignancies and 12% had solid tumors. The main reasons for ICU admission were acute respiratory failure (70%), acute renal failure (43%), shock (19%), and hepatic failure (12%). ICU and hospital mortality were 36% and 41%, respectively. Therefore, emergency anticancer therapy in selected cancer patients was feasible [6]. Since then, reports on EAT have gradually increased. In a recently review, Lafarge et al. systematically summarized the main hematological complications requiring EAT, including tumor lysis syndrome, disseminated intravascular coagulation or malignancy-associated hemophagocytic lymphohistiocytosis, hyperleukocytosis, and plasma hyperviscosity syndrome [2].

In patients who developed hyperkalemia, severe hyperphosphatemia or acidosis, and fluid overload unresponsive to diuretic therapy after chemotherapy, the early renal replacement therapy (RRT) should be considered [7]. Darmon et al reported that hospital and 6-month mortality rates were significantly lower in patients without acute renal injury than in the tumor lysis syndrome-related renal injury group. Therefore, preventing progression to acute renal injury in patients with tumor lysis syndrome is crucial to decrease mortality [8]. Zafrani et al proposed a strategy that in high risk tumor lysis syndrome patients, cytotoxic chemotherapy or debulking strategy should be given in the facility with ready access to renal replacement treatment [9].

Overt DIC was observed in approximately 1/3 of patients with acute leukemia [10]. It is recommended that the presence of DIC should not preclude or delay anti-neoplastic therapy in patients with acute leukemia by the International Society on Thrombosis and Haemostasis (ISTH), the Hemostasis & Malignancy Subcommittee and the Perioperative & Critical Care Thrombosis and Hemostasis Subcommittee [11].

Arca et al reportd 162 patients with hemophagocytic lymphohistiocytosis including 75 patients with haematological malignancy-associated hemophagocytic lymphohistiocytosis. They found that usage of etoposide was associated of improved 30-d survival [12]. Therefore, it is reconmmended that for malignant-triggered hemophagocytic lymphohistiocytosis, if severe organ damage is imminent, dose-adjusted etoposide may be used prior to tumor-specific treatment [13].

Hyperleukocytosis is present in 20% to 30% of patients with acute leukemia, and leukostasis is a hyper-viscosity syndrome encountered in up to 30% of hyperleukocytic acute leukemia [2]. Stahl et al reported 779 acute leukemia patients with hyperleukocytosis. Of them, 484 patients were admitted to ICU. They found that pulmonary involvement is the most common manifestation of leukostasis, occurring in over 40% of cases, and brain nvolvement occurs in over 30% of patients, and leukostasis is associated with increased 30-day mortality [14]. It is recommended that either hydroxyurea or planned induction therapy is administered without dealy [15].

EAT is also feasible in selected patients with cancer and acute respiratory failure. In a study involving 497 acute respiratory failure patients with cancer, after consulatation between intensivist and oncologist, all patients received EAT with a hospital mortality of 47% [3]. Louie et al. restrospectively collected data on 26 patients with malignant airway obstruction who received emergency radiotherapy. Of these, 7 patients were extubated [16]. We designed the questionnaire according to the above results that demonstrated survival benefit of EAT in selected patients with cancer.

Zerbib et al. reported that 136 patients with solid tumors received urgent chemotherapy. Of these, 81% had acute respiratory failure. All patients received EAT and ICU mortality was 37%. Multivariable analysis demonstrated that the presense of small cell lung cancer type was associated with improved hospital survival [17].

An increasing number of ICU physicians are accepting the concept of EAT. In a recent study, 84 patients received oncologic therapies in an oncologic ICU, 56% patients received EAT and 34.5% received oncologic therapies when their scheduled therapy was due [18]. In addition to oncologic ICU, EAT has also been reported in mixed ICUs. Lee et al. reported the use of tyrosine kinase inhibitors in 35 patients with non-small cell lung cancer harboring sensitizing epidermal growth factor receptor mutations who requeing mechanical ventilator in the ICU[19]. The 28-day ICU survival rate was 77%, and the authors concluded that tyrosine kinase inhibitors were useful for these patients suffering from respiratory failure and undergoing mechanical ventilation.

In our study, we also observed variations in the MDT composition. A previous study reported that organizational aspects, the presence of clinical pharmacists in the ICU, and close collaboration between oncologists and ICU teams improve mortality and resource use in critically ill patients with cancer (Odds Ratio: 0.66) [20]. In another study, intensivist involvement in lung cancer care was associated with significantly reduced hospital mortality (Odd Ratio : 0.42) [21]. Therefore, the MDT for a critically ill patient with cancer should include, at a minimum, an intensivist, an oncologis and a pharmacist.

The study had several limitations. First, EAT benefit s only selected patients with cancer. However, the optimal population requires further validation in large multicenter studies. Second, the sample size was relatively small. Nethereless, the responders included academic and mixed ICU intensivists, who represented attitudes of most ICU physicians in China.

In conclusion, this survey provided first insights of emergency anticancer therapy attitudes in intensivist. Dispite controversies remains regarding the MDT composition, there is a general positive attitude toward emergency anticancer therapy in intensive care medicine for critically ill patients with cancer-related organ dysfunctions.
